# A Cognitive Profile of Obesity and Its Translation into New Interventions

**DOI:** 10.3389/fpsyg.2015.01807

**Published:** 2015-11-27

**Authors:** Anita Jansen, Katrijn Houben, Anne Roefs

**Affiliations:** Department of Clinical Psychological Science, Faculty of Psychology and Neuroscience, Maastricht University, Maastricht, Netherlands

**Keywords:** obesity, cue reactivity, extinction, executive functions, inhibition, working memory, immediate reward, attention bias

## Abstract

Change your lifestyle: decrease your energy intake and increase your energy expenditure, is what obesity experts tell people who need to lose weight. Though the advice might be correct, it appears to be extremely difficult to change one’s lifestyle. Unhealthy habits usually are ingrained and hard to change, especially for people with an “obese cognitive profile.” Knowledge of the cognitive mechanisms that maintain unhealthy eating habits is necessary for the development of interventions that can change behavior effectively. This paper discusses some cognitive processes that might maintain unhealthy eating habits and make healthier eating difficult, like increased food cue reactivity, weak executive skills and attention bias. An effort is also done to translate these basic scientific findings into new interventions which aim to tackle the sabotaging cognitive processes. Preliminary studies into the effectiveness of these interventions, if available, are presented.

## Introduction

*Genes load the gun, the environment pulls the trigger* (Bray, 2004) is a frequently cited one-liner when experts discuss obesity. The one-liner refers to obesity following almost deterministically from an interaction between a specific genetic predisposition and the current “obesogenic” environment. It is argued that our old genes cannot handle such a modern toxic culture as the current one, in which the food industry aggressively markets their cheap calories, which are available in abundance. The calories are not only easy-to-get, they are also highly rewarding: fast foods are processed foods, saturated in fat, sugars and/or salt, which is what most people like. They are strongly wanted reinforcers (Epstein and Leddy, 2006; Giesen et al., 2009) which easily elicit reward-driven or “hedonic” eating, meaning that the eating is fully driven by pleasure instead of hunger or an energy deficit (Appelhans, 2009). The ultimate cause of obesity, or excess body fat, is calorie intake exceeding calorie expenditure (Wood, 2006). In addition to the huge availability of high-caloric foods, exercise is severely discouraged by technological developments nowadays: almost everything can be done by car, computer or smartphone.

Though obesity is considered a normal response to modern society, especially in genetically predisposed individuals (Wadden et al., 2002), it is a very serious global health problem. Its prevalence currently is about 35% in the USA (Center for Disease Control and Prevention, 2015), 10–30% in European Union countries and below 10% in Asia (World Health Organisation Europe, 2015) though a marked increase in its prevalence is noticed in Asia (Ramachandran and Snehalatha, 2010). Obesity is health threatening; it is associated with multiple medical conditions, seriously increased mortality, increased depression and decreased quality of life (Jia and Lubetkin, 2010). It also brings along high medical costs and a significant loss in productivity as a result of increased sick leaves (Neovius et al., 2010).

For many decades, a wealth of research is done into the genetic and biomedical characteristics of obesity on the one hand, and into the environment facilitating overeating on the other hand. Though it is generally argued that an unfortunate interaction of genes and environments predisposes to obesity, the ultimate solution that is most frequently given by experts to reduce or prevent obesity is relatively simple: Change your lifestyle. The environment, including the food industry, is difficult to change and so are genes. Health and food experts, like dieticians, nutritionists, physicians, and health insurance companies therefore stress the importance of healthier diets and a less sedentary life style. Their assumption however is that if people know what to do, they can do it. But that is a mistake. Many obese people do know that they should eat less and healthier and that they should exercise more, but most of them do not succeed in changing their behavior for a longer period of time. The majority of dieting attempts is unsuccessful in the long run: many dieters are able to lose some weight in the short term, but most of them regain their lost weight quickly and they frequently end up with more kilos than initially lost (Wing and Hill, 2001; Wing and Phelan, 2005). It is estimated that less than 20% of the obese are capable to reduce to a healthy weight in the long run (Mann et al., 2007). Lifestyle intervention studies aiming at weight loss often show statistically significant changes in BMI, but these changes are usually small (around 5%) and short-lived (Jeffery et al., 2000; Curioni and Lourenco, 2005; Franz et al., 2007; Powell et al., 2007; Barte et al., 2010; Kirk et al., 2012; Wadden et al., 2014). Lowe (2015) points to high correlations between starting BMI’s (before weight loss) and subsequent BMI’s at follow ups after dieting. Though participants might experience positive health consequences of relatively small weight losses, many of them are still obese after a lifestyle intervention. Why are people so unsuccessful in dieting and losing weight? Why is it so difficult to resist the “toxic” obesogenic environment?

All diets work when one sticks to them (Thomas et al., 2008; Johnston et al., 2014). People who experience difficulty in changing their eating habits often mention that it is difficult to comply with the dietary recommendations. “I *do* know it, but I can’t,” “I do succeed for some days but then, in the evening, it goes wrong again,” “I am insatiable,” “when I do eat something that is forbidden, it feels like my diet is ruined, and I might as well go on eating. It usually takes days before I am back on track again.” The issue is that knowledge of healthy eating and even support of a lifestyle coach is not enough to change one’s eating behavior. The most important determinant of weight loss is adherence to a calorie restricted diet (Hall et al., 2015) and adherence is easier when ingrained bad habits change (Rothman et al., 2009).

What do we know about the mechanisms that maintain ingrained unhealthy eating habits and make adherence to the new diet difficult? This article presents an overview of some cognitive processes that might sabotage successful dieting. Further, an effort is done to translate the scientific findings into new cognitive-behavioral intervention modules which aim to tackle the sabotaging cognitive processes and could help sticking to a diet. Preliminary studies into the effectiveness of these interventions, if available, will also be discussed.

## Acquisition of Food Cravings

Desires to eat and food cravings can easily be learned. Current frequent exposures to highly palatable food make the number of daily possibilities to associate cues and contexts with tasty eating almost endless (Kessler, 2009; Rothman et al., 2009; Bouton, 2011). Any time food is ingested, there is an opportunity to associate the eating with cues that are present at the time (Jansen, 1998; Bouton, 2011). A large number of animal studies have shown that the physiological responses brought about by food intake (e.g., insulin release, blood sugar increase, and salivation) can be brought under the control of any stimulus predictive of the eating, like odors, time of the day, the seeing, smelling and tasting of food and also contexts (Jansen, 1998; Bouton, 2011; Jansen et al., 2011a).

The learning of such an association between cues or contexts and intake basically is a form of classical conditioning; the cues and/or contexts that are associated with intake will become signals (conditioned stimuli) for consumption (unconditioned stimulus). Animal studies show that rats consume significantly more of less preferred foods (chow) when exposed to context cues that were earlier paired with the intake of highly palatable foods (Boggiano et al., 2009). The authors conclude that context-cues associated with palatable food intake might drive overeating in rats, even when they are sated and when the food is less-preferred. They also report that cue-conditioned overeating is quickly learned and particularly strong when the taste of a palatable food is used to make cue-food associations (Boggiano et al., 2009).

In humans, it likewise appears to be relatively easy to learn cued eating desires by means of classical conditioning procedures (Van Gucht et al., 2008; Papachristou et al., 2013; Van den Akker et al., 2013, 2014, 2015; Bongers and Jansen, 2015; Bongers et al., 2015). After the learning of a stimulus predicting intake, the mere presence of the food-predictive stimulus is sufficient to elicit eating expectations and desires. The daily life analogy is that if one eats chocolate every evening after dinner around eight o’clock, one will soon experience a desire for chocolate after dinner just before or around eight. Even when one is satiated, a signal that predicts consumption is able to elicit food desires, e.g., just thinking of how delicious the dessert would taste might make people feel “hungry” and eat, even when they had a large meal, which causes cued eating to increase the risk of overeating (Jansen et al., 2011a). Also eating-related emotions can elicit cued overeating, sometimes referred to as emotional eating (Bongers and Jansen, 2015; Bongers et al., 2015).

The responding to cues that signal the availability of tasty foods, is called food cue reactivity. These responses can be physiological, like a salivation response or neural activation, or psychological, like a desire to eat or craving, and might easily lead to cued (over)eating and—in the end—weight gain (Boswell and Kober, in press). Increased cue reactivity and cued overeating after the smelling of tasty foods during short exposures is generally found in healthy adults, it is a normal response (Nederkoorn et al., 2000; Ferriday and Brunstrom, 2011; Jansen et al., 2011b). However, cue reactivity is significantly stronger in patients with bulimia nervosa (Vögele and Florin, 1997; Neudeck et al., 2001; Legenbauer et al., 2004; Nederkoorn et al., 2004), and obese people (Tetley et al., 2009; Ferriday and Brunstrom, 2011) compared to lean adults without eating disorders. Overweight children demonstrated cued overeating while lean children did not and, only in the overweight children, the amount eaten correlated strongly with increased salivary responding during exposure to the food cues (*r* = 0.62; Jansen et al., 2003). To conclude, food cue reactivity appears to be stronger in eating disordered and obese people and the food cue reactivity motivates eating, also in the absence of hunger and in excess of calories needed.

Though food cue reactivity shows a strong learning component (classical conditioning), it also has a genetic component. About 67% of the variability in BMI has a genetic basis, of which only about 10% is related to metabolic processes (Ravussin and Bogardus, 2000; Llewellyn and Wardle, 2015), while the largest part of the genetic predisposition for obesity is reflected in a hyperresponsivity to tasty food cues—or food cue reactivity (Carnell et al., 2008; Llewellyn et al., 2010). It would be of interest to study whether a genetic contribution to food cue reactivity and hyperresponsivity to tasty food cues also means facilitated acquisition of associations between food cues and eating.

To sum up, increased reactivity to food related cues, including emotions, thoughts and contexts, might predict cued overeating. Cue reactivity, meaning increased desires to eat or food cravings in response to cues signaling intake, easily sabotages healthy eating and might make weight loss and its maintenance more difficult.

### Translation to Intervention Module

Reduced reactivity to tempting food cues, i.e., less appetitive responding to palatable food cues, might make healthy and controlled eating easier (Jansen et al., 2011a). Indeed, successful dieters report to especially refrain from eating palatable high calorie fat foods; they say they are continually strict dieters who show little variety in their diets (Wing and Hill, 2001; Gorin et al., 2004; Phelan et al., 2008). In line with the cue reactivity model, it was found that successful post-obese dieters showed significantly less cue reactivity (salivation) during food cue exposure compared to unsuccessful obese dieters (Jansen et al., 2010) and a normalized habituation of the salivation response (Bond et al., 2010). So the critical question is: how can we reduce or extinguish cue reactivity?

Exposure therapy is a well-known behavioral intervention that is applied in anxiety disorders for many decades and appears to be effective in reducing fear and avoidance behavior. A translation to addictive disorders (e.g., Drummond and Glautier, 1994) and eating disorders (e.g., Jansen et al., 1989) was made several decades ago. The food cue exposure with response prevention aims to extinguish food cue reactivity and to decrease cued overeating (Jansen et al., 1992, 2011a; Jansen, 1998; Havermans and Jansen, 2003). Food cue exposure was different from existing exposures at that moment, which aimed to reduce anxiety, for example the anxiety that is associated with eating a large amount of high calorie foods while vomiting was prevented (see Schmidt and Marks, 1988).

Food cue exposure aims to break the bond between cues and overeating by prolonged and repeated non-reinforced exposure to the cues that predict overeating and its aim was not to establish a reduction in anxiety but a reduction in the desire to eat. During the exposure one is confronted with the cues that signal unhealthy eating, like the sight, smell and taste of one’s favorite foods, and overeating related contexts like specific places or situations, emotions, thoughts and attributes. The food is touched during the exposure, one grabs it, holds it to the nose and smells it intensely and prolonged. Taste usually is a straightforward signal for intake and elicits desires; a very little bite to experience the taste is stimulated, without further eating. In this way it is gradually learned that the cues not always signal unhealthy and/or uncontrolled eating, they ultimately start predicting no-intake or no-eating (and presumably learned inhibition). The exposure lasts for about an hour and will, in the end, lead to decreased cue reactivity (decreased desires to eat) and increased inhibition skills (no eating) because one gradually learns that the cues can also predict no eating. A well-controlled experimental study (Schyns et al., in press) and some small-scaled pilot studies suggest that food cue exposure can be quite effective in reducing food cravings, overeating and binge eating (Jansen et al., 1992; Toro et al., 2003; Martinez-Mallén et al., 2007; Boutelle et al., 2011). A recent neuroimaging study showed that prolonged food cue exposure (smelling) without eating leads to reduced reward-related activity in the brain (Frankort et al., 2014).

## Executive Functioning

Executive functioning refers to a set of skills and processes that are concerned with managing oneself and the use of one’s cognitive resources in order to achieve a goal or to perform goal-directed actions (Baddeley, 1986; Norman and Shallice, 1986; Miyake et al., 2000). Three main skills are inhibition (the ability to stop one’s behavior at the appropriate time, the ability to resist impulses and temptations, thereby disabling goal-oriented actions), shift (the ability to think flexibly in order to respond appropriately to a situation), and working memory (the capacity to hold information in memory to complete a task). When executive functions are impaired, automatic impulses (for example: eat!) are not adequately handled, and undesired behavior (e.g., unhealthy or uncontrolled eating) may follow (Hofmann et al., 2012). Excellent executive functioning skills are also referred to as high self-control. Executive function deficits were found to be associated with addictions and overeating (Verdejo-García et al., 2008) and recent studies into the executive functioning of obese people also show dysfunctional executive profiles in obese people (Fagundo et al., 2012; Reinert et al., 2013).

*Response inhibition* is the executive functioning skills that has been studied most frequently in obesity. It refers to the ability to *not* perform a response, to overrule automatic intentions to directly respond to stimuli without thinking, to refrain from direct acting. A robust finding is that obesity is associated with less effective inhibitory control (Nederkoorn et al., 2006a,b,c, 2009; Guerrieri et al., 2008). Moreover, providing evidence for a *causal* relationship between lack of inhibition and overeating, it was found that an experimental induction of a disinhibitive response style in healthy normal weight participants led to an increase in food intake (Guerrieri et al., 2009). But the reverse may be true as well, that is, overeating can lead to decreased inhibition (Martin and Davidson, 2014). Possibly, both strengthen each other.

Though some studies suggest that the insufficient response inhibition of obese people is a more general behavioral trait, i.e., not specifically related to food and eating, recent studies argue that it is specifically related to food-related responding (Batterink et al., 2010; Mobbs et al., 2011; Nederkoorn et al., 2012; Houben et al., 2014). Overeating is suggested to follow from a lack of inhibitory control over in particular the hedonic, appetitive system (see, e.g., Appelhans, 2009; Houben and Jansen, 2014, 2015; Houben et al., 2014) which makes it difficult to refrain from (over)eating in tempting situations.

Decreased inhibition abilities are also related to unsuccessful dieting: they hinder weight loss and its maintenance. Two studies show that obese children with less inhibition skills (measured before treatment) lost less weight during a cognitive behavioral weight loss intervention compared to obese children with more inhibition skills (Nederkoorn et al., 2006b; Kulendran et al., 2014). However, another study found that poor inhibition skills actually predicted better weight loss in adolescents (Pauli-Pott et al., 2010). The idea of increased inhibitory control in successful dieters and decreased inhibitory control in obesity is indirectly supported by the demonstration of decreased prefrontal cortex (PFC) metabolism in obesity (Volkow et al., 2008, 2011), whereas increased PFC activity was observed in successful dieters (DelParigi et al., 2007; Sweet et al., 2012).

To conclude, decreasing levels of inhibition skills might facilitate overeating in tempting situations, thereby reducing the effect of weight loss interventions and increasing the risk of relapse after successful weight loss, while increasing levels of inhibition skills might facilitate successful weight loss and its maintenance.

### Translation to Intervention Module

For obesity is related to deficits in executive functioning, it is of interest to study whether improvement of executive functioning is possible, and whether this improvement makes it easier to refrain from unhealthy eating. Some experimental studies tested the effects of executive functioning training in obese people. Indeed, the experimental priming of inhibition and control led to improved inhibition skills and decreased subsequent consumption (Guerrieri et al., 2009, 2012). Also, participants who learned to respond to healthy foods while inhibiting responses toward unhealthy foods, significantly reduced their snack intake thereafter (Houben and Jansen, 2011, 2015; Veling et al., 2011, 2013; Houben et al., 2014; Lawrence et al., 2015b; but see also Allom and Mullan, 2015) and some studies even show reduced body weight following inhibition training (Veling et al., 2014; Lawrence et al., 2015a). Comparable training effects were found for excessive drinking behavior: response inhibition training decreased alcohol consumption (Houben et al., 2011a, 2012; Bowley et al., 2013; Jones and Field, 2013).

Also when working memory is weak, one’s behavior is more strongly guided by impulses. Would it also be effective to train one’s working memory, to increase cognitive control and to show better resistance to temptations? A working memory training in heavy drinkers appeared to be effective in improving working memory and decreasing alcohol intake (Houben et al., 2011b). In obese children, the training of inhibition skills together with the training of working memory led to a significantly slower relapse (weight regain) at the 8-week follow up, though this effect had disappeared at 12 weeks (Verbeken et al., 2013). To the best of our knowledge, as of yet, no studies have been published into interventions that tested the isolated effect of working memory training in obesity.

To conclude, although interventions that aim to improve executive functioning are still in their infancy, preliminary results seem promising. It is useful to further study ways that promote better executive functioning in obese people: weight loss will probably be easier and more successful when one shows better self-regulation and is less led by impulsive responding.

## Immediate Rewards

Eating is inseparably associated with reward. Food is a potent natural reward, especially when it is high in calories, fat, salt and sugar. It is argued that the reward system plays a key role in the development of obesity (see, e.g., Appelhans et al., 2016). Weight control would imply that one does not lose oneself in immediate temptations (eating the rewarding foods) but that one is directed by long-term healthy goals (weight loss or its maintenance). Individuals differ in their ability to resist temptations and immediate rewards (Appelhans et al., 2016). Some people find it difficult to wait for later rewards and have a strong drive to get rewards directly. This so-called inability to delay gratification was demonstrated repeatedly in obesity (e.g., Davis et al., 2004a,b; Franken and Muris, 2005; Davis et al., 2007; Weller et al., 2008; Daniel et al., 2013a). The desire to experience direct pleasure or reinforcement is also present when the preferred direct reinforcement is smaller than a delayed larger reinforcement, i.e., delay discounting. An example is the decision to eat the tasty foods now at the expense of long term weight control, though delay discounting is also found in the obese when it is not related to eating: obese children and adults gamble longer for immediate rewards even when the chances of winning decrease and stopping would lead to greater gain (e.g., Dougherty et al., 2003; Davis et al., 2004b; Nederkoorn et al., 2006a,c; Epstein et al., 2010, 2014).

To conclude, the striving for immediate rewards at the expense of long term weight control reduces the effect of life style interventions, while the ability to delay gratification might facilitate successful weight loss and its maintenance.

### Translation to Intervention Module

An intervention that improves the ability to delay gratifications is needed. A better ability to delay rewards might reduce impulsive uncontrolled overeating and facilitate weight control (Dassen et al., 2015). It is possible that food cue exposure and inhibition training improve the ability to delay gratification as well. Recently, there have been some studies that investigated interventions based on episodic future thinking that directly tackled delay discounting to reduce food intake (Daniel et al., 2013b, 2015; Dassen et al., 2016). Episodic future thinking is a strategy to shift one’s preference from immediate gratification to delayed rewards (Peters and Büchel, 2010) and refers to the projection of oneself forward in time and to pre-experience future events (Atance and O’Neill, 2001), resulting in an increased choice of delayed rewards (Koffarnus et al., 2013). Episodic future thinking reduced delay discounting and also energy intake compared to an non-future episodic thinking control group (Daniel et al., 2013b, 2015; Dassen et al., 2016), making future episodic thinking training a promising intervention to reduce impulsive overeating.

## Attention Bias

The increased attractiveness of high calorie foods was found to be reflected in biased attentional processing of food cues in obese people (Braet and Crombez, 2003; Castellanos et al., 2009; Werthmann et al., 2011) A food-related attention bias (AB) refers to selective attentional processing of food cues, including increased attention for, and interference by, food cues compared to other cues but also the avoidance of food cues relative to other cues might reflect an AB. Werthmann et al. (2011) studied the course of attention during exposure to high calorie food cues vs. low calorie and neutral cues in obese vs. normal weight participants using an eye tracker. An AB for high fat foods was found in obese people. More specifically, the obese participants showed more frequent initial orientation towards the high fat foods and this initial orientation was followed by reduced attention for the high fat foods, suggesting an approach—avoidance ambivalence in obese people (Werthmann et al., 2011).

However, several studies do not show attention biases in obese people (see for an overview of these studies: Roefs et al., 2015). The authors argue that AB is state-dependent. That is, obese people may not always have an attentional bias toward food, which may make the restriction of food intake more difficult, but only when their mindset is focused on hedonics (Roefs et al., 2015; Werthmann et al., 2015a). One study found that a bias toward high-calorie foods is especially related to food cravings in a sample of female college students (Werthmann et al., 2013). Other studies found that induced food cravings lead to an AB in high trait chocolate cravers (Kemps and Tiggemann, 2009; Smeets et al., 2009), and that induced satiety in a sample of hungry participants leads to a decrease in AB (Di Pellegrino et al., 2011). The other way around, an induced AB for high calorie foods (chocolate) elicits food cravings in undergraduate students (Kemps et al., 2014; Werthmann et al., 2014), and undergraduate students trained to attend to healthy foods eat relatively more healthy foods afterwards (Kakoschke et al., 2014). Initial orientation towards high calorie foods in obese participants was found to be positively associated with subjectively experienced cravings and significantly higher intake during a bogus taste test (Werthmann et al., 2011). In a recent study, the same authors showed that initial orientation bias predicted reduced weight-loss in obese children that participate in a lifestyle treatment (Werthmann et al., 2015b).

### Translation to Intervention Module

There is some evidence that biased attention toward food predicts the strength of experienced cravings, the amount eaten, and even the amount of weight gained in obesity. It could be wise to retrain attention in such a way that it is not biased to high calorie foods anymore. Studies into the effect of AB modification training are promising: they demonstrate that training attention away from high calorie/unhealthy foods favoring attention for low calorie/healthy foods or neutral stimuli, reduces food cravings and food intake (Boutelle et al., 2014; Kakoschke et al., 2014; Kemps et al., 2014; Werthmann et al., 2014).

## Conclusion

**FIGURE 1:**
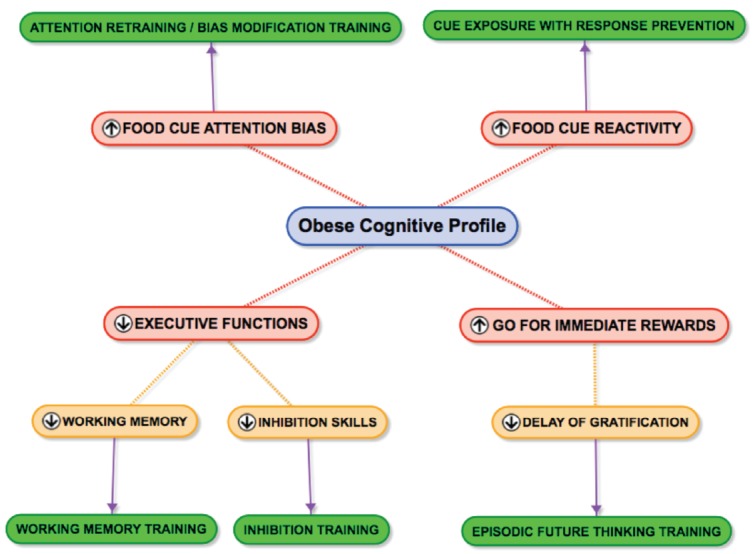
**The obese cognitive profile.** Some cognitive mechanisms (in red) maintaining overeating and matched intervention modules (in green) to tackle the sabotaging cognitive processes. For the sake of simplicity arrows are only drawn between the specific process and the matched training module. Reality probably is more complex: the cognitive processes are interrelated and might influence each other (e.g., weak executive skills and drive for immediate rewards might increase food cue reactivity) and a specific training module might not only influence the specific process but also have an effect on other cognitive processes (e.g., extinction training might reduce biased attention and disinhibition).

To conclude, while genes and the environment may load the gun, it seems that cognitive processes pull the trigger: obesity mainly is a behavioral and cognitive condition. The current environment is especially obesogenic for people who are strongly food cue reactive, sensitive to immediate rewards and have weak executive skills. Experts advice them to change their lifestyles: They should eat less and healthier and they should exercise more frequently. Though the advice essentially is correct, it appears to be very difficult to change one’s lifestyle, especially for food cue reactive people with weak executive skills who are sensitive to immediate rewards. Insight into the cognitive maintaining mechanisms that are associated with food cue reactivity, weak executive skills and reward sensitivity are necessary for effective behavior change. It is argued that unhealthy eating habits can be changed by interventions that tackle these cognitive maintenance mechanisms. This paper discussed some cognitive maintenance mechanisms and suggests some new intervention modules (see Figure 1): extinction training or cue exposure with response prevention, training of executive functions like inhibition, working memory and delay of gratification, cognitive restructuring and AB modification training. A simple straightforward line from a specific process to a matched training module is drawn, though reality surely is more complex: the diverse cognitive processes are interrelated and influence each other (e.g., weak executive skills and reward sensitivity might increase food cue reactivity) and a specific training module might not only influence specific process but might generalize its effects to other cognitive processes (e.g., extinction training might reduce biased attention and disinhibition). It means that there is room for lots of new research.

## Author Contributions

All authors contributed equally to this work.

### Conflict of Interest Statement

The authors declare that the research was conducted in the absence of any commercial or financial relationships that could be construed as a potential conflict of interest.
